# RNA-Binding Proteins as Regulators of Migration, Invasion and Metastasis in Oral Squamous Cell Carcinoma

**DOI:** 10.3390/ijms21186835

**Published:** 2020-09-17

**Authors:** Jonas Weiße, Julia Rosemann, Vanessa Krauspe, Matthias Kappler, Alexander W. Eckert, Monika Haemmerle, Tony Gutschner

**Affiliations:** 1 Junior Research Group ‘RNA Biology and Pathogenesis’, Medical Faculty, Martin-Luther University Halle-Wittenberg, 06120 Halle/Saale, Germany; jonas.weisse@uk-halle.de (J.W.); julia.rosemann@uk-halle.de (J.R.); vanessa.krauspe@student.uni-halle.de (V.K.); 2 Department of Oral and Maxillofacial Plastic Surgery, Medical Faculty, Martin Luther University Halle-Wittenberg, 06120 Halle (Saale), Germany; matthias.kappler@uk-halle.de; 3 Department of Cranio Maxillofacial Surgery, Paracelsus Medical University, 90471 Nuremberg, Germany; alexander.eckert@klinikum-nuernberg.de; 4 Institute of Pathology, Section for Experimental Pathology, Medical Faculty, Martin-Luther University Halle-Wittenberg, 06120 Halle/Saale, Germany; monika.haemmerle@uk-halle.de

**Keywords:** HNSCC, oral cancer, OSCC, RBP, IGF2BP3, LIN28, HuR, METTL3, DDX3

## Abstract

Nearly 7.5% of all human protein-coding genes have been assigned to the class of RNA-binding proteins (RBPs), and over the past decade, RBPs have been increasingly recognized as important regulators of molecular and cellular homeostasis. RBPs regulate the post-transcriptional processing of their target RNAs, i.e., alternative splicing, polyadenylation, stability and turnover, localization, or translation as well as editing and chemical modification, thereby tuning gene expression programs of diverse cellular processes such as cell survival and malignant spread. Importantly, metastases are the major cause of cancer-associated deaths in general, and particularly in oral cancers, which account for 2% of the global cancer mortality. However, the roles and architecture of RBPs and RBP-controlled expression networks during the diverse steps of the metastatic cascade are only incompletely understood. In this review, we will offer a brief overview about RBPs and their general contribution to post-transcriptional regulation of gene expression. Subsequently, we will highlight selected examples of RBPs that have been shown to play a role in oral cancer cell migration, invasion, and metastasis. Last but not least, we will present targeting strategies that have been developed to interfere with the function of some of these RBPs.

## 1. Introduction

Cancers of the lip, tongue and oral cavity were estimated to collectively account for approximately 355,000 newly diagnosed neoplasia and over 177,000 cancer deaths in 2018 ranking oral cancer on position 16 and 15 in terms of incidence and mortality, respectively [1,2]. Oral squamous cell carcinoma (OSCC), which account for more than 90% of all oral tumors develop from cells in the basal layer of the oral epithelium and originate from either altered stem cells or through dedifferentiation of early-stage differentiated cells [3,4,5]. Known risk factors of oral cancer include tobacco and alcohol abuse, consumption of areca nut products as well as human papilloma virus (HPV) infection [6,7,8]. Carcinogen exposure can trigger a complex multistep process characterized by an accumulation of genetic and epigenetic alterations that lead to genomic instability and loss of tumor suppressor genes (*TP53*, *CDKN2A*, *RB1*, *RBL1/2*) as well as activation of oncogenic signaling pathways including epithelial growth factor receptor (EGFR), phosphatidylinositol-3-kinase (PI3K)/AKT/mammalian target of rapamycin (mTOR), mitogen-activated protein kinase (MAPK), and Janus kinase/signal transducers and activators of transcription (JAK/STAT) that contribute to oral cancer progression [9,10]. In HPV-negative patients, more than 80% of the tumors harbor *TP53* loss-of-function mutations, which occur in early stages often combined with mutations in the Rb pathway [11,12]. In HPV-positive patients, p53 protein is degraded via HPV E6 and pRb via HPV E7. In addition, a high prevalence of inactivating mutations in *NOTCH1* have been identified suggesting a tumor-suppressive role of Notch signaling in head and neck squamous cell carcinomas (HNSCC) in general and OSCC in particular [11]. However, several lines of evidence are emerging and support the idea of a rather oncogenic function of the Notch1 pathway which might be an attractive target for treatment of HNSCC [13,14].

Identification of novel and high-confidence therapeutic targets in OSCC is an urgent need and despite significant advances in the diagnosis and treatment, the 5-year survival rate is ~60%, which decreases to ~30% for patients with advanced disease (https://seer.cancer.gov/csr/1975_2006/) [15,16]. One reason for this high mortality rate is the late diagnosis of OSCC when the cancer has already metastasized. In fact, at the time of diagnosis, over 50% of oral cancer patients in the United States have already developed regional or distant disease [17]. Moreover, OSCC has a high recurrence rate and frequently metastasizes to cervical lymph nodes with lymph node metastasis occurring in 40% of OSCC patients [18,19,20]. Lymph node involvement and extracapsular spread are strong prognostic factors [21,22]. The overall prevalence of distant metastasis in clinical studies commonly lies between 8–14% [18]. In contrast, autopsy studies revealed a 40–57% incidence of patients with distant metastasis [23,24,25]. The lung is the most frequent site for OSCC metastasis (~80%) followed by bone, liver and mediastinal nodes [18,24]. Once distant metastases are diagnosed, the median time to death is only 3.3 months [18]. Thus, understanding the mechanisms responsible for the malignant spread of oral cancer cell is key to develop effective therapeutics and extend the life of patients.

The development of metastasis is a multifactorial phenomenon, which, in the case of head and neck cancers, includes the following factors that significantly increase distant metastasis: extracapsular extension, location of the primary tumor in the hypopharynx, higher stage grouping, N classification, locoregional failure, including relapse and persistent disease [18]. HPV positivity was associated with less distant metastasis [18]. In addition, lymphovascular and perineural invasion have been suggested to affect distant metastasis and survival by some studies, yet their role in oral cancer remains controversial [18,26,27,28,29]. Importantly, our current understanding of the underlying molecular events and individual factors involved in the metastatic dissemination of oral cancer cells is still very limited. It is well-known that several steps along the invasion-metastasis cascade have to be taken by tumor cells in order to spread from the primary tumor site to an anatomically distant location [30]. First, the cells have to detach from the primary tumor and locally breach the basement membrane in order to invade the surrounding extracellular matrix and connective tissue. Next, the cancer cells have to intravasate the blood or lymphatic vessels and travel to distant anatomical sites where they extravasate from the vessels into the stroma of the metastatic site. Here, the tumor cells form micrometastases and eventually begin to expand and start their proliferative program to colonize the tissue and ultimately form macrometastases. Importantly, in order to be successful, tumor cells need to be able to tolerate and adapt to several different stress factors and changing environments. Hence, it is increasingly recognized that phenotypic plasticity, largely driven by epigenetic and transcriptional mechanisms, markedly influences the metastatic progression [31,32,33]. A well-studied and important source of plasticity of malignancy is epithelial- to-mesenchymal transition (EMT), an epigenetically controlled event that enables transitions of malignant cells between different phenotypic states that confer motility and enhance survival [5]. During EMT of cancer cells, which is promoted by an inflammatory immune response and the hypoxic microenvironment, cells lose their adhesiveness and apical-basal polarity, and undergo both cytoskeletal and signaling changes which enhances their ability to migrate and invade [5,34,35]. EMT and its underlying regulatory mechanisms have been extensively studied and a central role for EMT in the metastasis of several cancer types, including OSCC has been described [36,37]. However, some controversies regarding the contribution and significance of EMT to tumor progression exist and alternative mechanisms of migration have to be considered, also in combination, to capture the full spectrum of cell states, which is a prerequisite to develop effective anti-metastasis strategies [38,39,40].

Of note, cellular plasticity conferred by EMT and other mechanisms that lead to morphological and functional changes requires robust reprogramming of gene expression programs. For example, several thousand genes change their expression in a context-dependent manner during EMT [41]. These profound changes require multiple layers of regulation, from transcription, to post-transcriptional RNA processing, to translational and post-translational modifications. While transcriptional regulation by EMT-associated transcription factors, e.g., members of the zinc finger E-box binding homeobox (ZEB), SNAIL and TWIST families, is generally considered the most important mode of regulation, mounting evidence indicates that post-transcriptional events strongly contribute to the fine-tuning of EMT [42,43,44]. This fine-tuning can be achieved through the action of microRNAs (miRNAs) and long non-coding RNAs (lncRNAs) as well as RNA-binding proteins (RBPs) [45,46,47,48,49,50].

In this review, we will focus on RNA-binding proteins and we will offer a brief overview about their general contribution to post-transcriptional regulation of gene expression. Subsequently, we will highlight selected examples of RBPs that have been shown to play a role in oral cancer cell migration, invasion, and metastasis. Last but not least, we will present targeting strategies that have been developed to interfere with the function of some of these RBPs.

## 2. General Functions and Mechanisms of RNA-Binding Proteins

Over the past decade, RBPs have been increasingly recognized as important regulators of molecular and cellular homeostasis. RBPs regulate diverse cellular processes such as cell survival, pluripotency of embryonic stem cells, and immune cell function, as well as aid in the transition between cellular states in response to stimuli, e.g., during differentiation, cellular stress, or viral infection [51,52,53,54,55,56,57,58]. Nearly 7.5% of all human protein-coding genes have been assigned to the class of RBPs (1542). These annotations were initially based upon the presence of characteristic RNA binding domains as well as their association with polyadenylated RNA-containing protein complexes that have been purified using a poly(A)-capture strategy [59,60,61,62]. An updated annotation compiled a list of 1914 and 1393 RBPs expressed in human and murine cells [63]. In addition, a recently developed experimental strategy to explore the complete RNA-bound proteome identified 1207, 1239, and 1357 proteins in MCF7, HeLa and HEK293 cells, respectively. 858 proteins were shared by all three cell lines. This study finally arrived at an integrated human RNA-binding proteome comprised of 978 proteins (70%) of previous poly(A) interactomes and 775 proteins constituting a novel non-poly(A) interactome [64].

In order to fulfill their cellular tasks and to influence the fate of hundreds to thousands of transcripts RBPs interact with specific sequences or RNA secondary structure elements within their respective target RNA to regulate its post-transcriptional processing, i.e., its alternative splicing, polyadenylation, stability and turnover, localization, or translation as well as editing and chemical modification. Therefore, RBPs typically contain discrete domains for the purpose of binding RNA and more than 40 RNA-binding domains (RBDs) have been described [59]. Importantly, RBDs are quite small (less than 100 amino acids) and utilize only a fraction of their residues to directly interact with RNA via hydrogen bonds, stacking interactions, and other weaker interactions. To achieve specificity multiple binding domains often co-exist within one RBP, thereby enhancing specific RNA binding [65]. Furthermore, linkers or intrinsically disordered regions between individual domains have been shown to mediate important RNA contacts as well, and the flexibility of linkers can determine whether adjacent RNA-binding domains bind independently or cooperatively [66]. Intriguingly, many of the recently identified RBPs, including a large group of metabolic enzymes, lack conventional RBDs and have no established connection to RNA processing events [67]. Hence, the exact nature and functional consequences of many of these RNA-protein interactions remain largely unknown. Nevertheless, an analysis of the genomic distribution of RBP binding motifs within their target transcripts revealed a large proportion of these motifs to be localized within the non-coding parts of the human genome, especially within introns and 3′ untranslated regions (UTRs) of messenger RNAs (mRNAs) [68,69]. From a functional point of view such a distribution of binding motifs makes sense, because these non-coding regions are less constrained in protein binding than the coding regions and 5′UTRs, in which excessive protein binding might interfere with function, e.g., translation. Importantly, RBPs execute their regulatory functions on both, coding and non-coding RNAs (ncRNAs), and might cooperate or compete with other post-transcriptional regulators, e.g., miRNAs, or might even be subjected to post-transcriptional control themselves. For example, association of proteins with lncRNAs can affect the localization and stability of both, the non-coding transcript as well as the protein. These interactions are of functional relevance for diverse cellular processes including, but not limited to transcriptional and epigenetic gene expression control, splicing, DNA damage signaling, tissue homeostasis and differentiation as well as cancer cell invasion and metastasis [70,71,72,73,74,75,76,77]. Furthermore, recent studies have identified several RBPs that regulate the processing and biogenesis of miRNAs and their precursors [78,79]. Because miRNAs preferentially target the 3′UTRs within mRNAs, it is more and more realized that there might be some competition or even cooperation between RBPs and miRNAs as exemplified by the well-characterized oncofetal RBP Insulin-like growth factor 2 mRNA-binding protein 1 (IGF2BP1), which was shown to enhance the expression of oncogenic factors by interfering with miRNA-targeting [58,80]. The opposite was observed for tristetraprolin (TTP) and human antigen R (HuR), which cooperate with miR-16 or let-7, respectively, by recruiting target transcripts to the RNA-induced silencing complex (RISC) thereby enhancing miRNA-mediated gene silencing [81,82]. Intriguingly, in the context of 3′UTR-dependent gene regulation, it was recently shown that the assembly of many protein complexes in eukaryotic cells occurs co-translationally whereat proteins of the same complex support each other’s three-dimensional folding [83]. While the underlying molecular mechanism(s) are largely unclear, it has been suggested that RBPs, through their binding to the 3′UTRs, might play a crucial role to enable proximity of two subunits near translating ribosomes [84]. This finding is well in line with other recent studies that established an important role for individual 3′UTRs in mediating protein-protein interactions thereby regulating diverse protein features, including protein complex formation, posttranslational modifications as well as protein conformation in a coding-sequence independent manner. Importantly, RBPs and their binding to the respective 3′UTRs was shown to be necessary for the 3′UTR-mediated effects [85,86,87,88].

**Figure 1:**
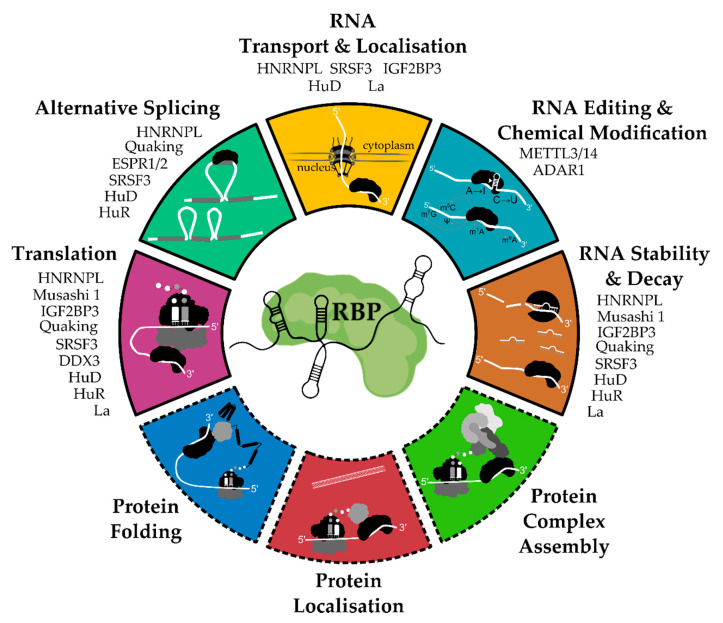
Functional hallmarks of RNA-binding proteins (RBPs). RBPs are important post-transcriptional regulators of gene expression. They are well-known to regulate RNA translation, splicing, transport, editing and chemical modification as well as turnover. Moreover, emerging functions of RBPs in co-translational protein complex assembly, protein localization and protein folding have been recently suggested. Hence, deregulated RBP activities can have broad effects on cellular homeostasis and are associated with several human diseases. Oral cancer-associated RBPs highlighted in this article have been assigned to relevant hallmark functionalities based on their established roles in human cancers as well as developmental processes.

We have summarized the classical as well as emerging hallmark functions of RBPs in Figure 1. Given their widespread impact on gene expression and signaling networks, mutations or defects in the expression or localization of RBPs can cause a broad range of diseases, e.g., neurodegeneration, obesity, hypertension, and cancer [55,89,90,91,92,93,94,95,96]. In the next paragraph, we will highlight selected examples of RBPs that have been shown to contribute to the malignancy of oral cancer, especially its metastatic features.

## 3. RBPs Associated with Migration, Invasion, and Metastasis in OSCC

As mentioned earlier metastases are the major cause of cancer-associated death. However, the role and architecture of RBPs and RBP-controlled expression networks in the diverse steps of the metastatic cascade is only incompletely understood. Here, we introduce some examples of RBPs whose association with the malignant spread of oral cancer cells has been recently established.

### 3.1. ADAR1

The adenosine deaminase acting on RNA 1 (ADAR1) protein is involved in adenosine-to-inosine (A-to-I) editing in double-stranded RNA and has been implicated in several human cancers [97,98]. Because inosines are read as guanosines (G), editing can enhance the complexity of the transcriptome and re-coding of the genetic information by the generation of non-synonymous codon changes or alternative splicing. RNA editing can also affect targeting or disrupt maturation of miRNAs [99]. In fact, a comparative whole genome expression microarray analysis revealed that ADAR1 fundamentally regulates miRNA processing, which was suggested to largely happen in an RNA binding–dependent, yet RNA editing–independent manner by regulating Dicer expression [100]. Another study could show that ADAR1 forms a complex with Dicer through direct protein-protein interactions which increases the maximum rate of pre-miRNA cleavage and facilitates RISC loading of mature miRNAs [101]. In line with this, a recent study could show that ADAR1 physically interacts with Dicer and promotes the maturation of oncogenic miRNAs in OSCC [102]. Moreover, the authors could show that high levels of ADAR1 in OSCC tissues are associated with primary tumor size, lymph node metastasis, stage as well as overall and disease-free survival. On the cellular level, ADAR1 was shown to promote proliferation, growth and stemness of OSCC cell lines. Moreover, overexpression of ADAR1 enhanced the migration and invasion phenotype of the cells and it was suggested that ADAR1 is involved in the EMT process of OSCC [102]. However, the function of ADAR1 and its clinical relevance in OSCC might depend on the ADAR1 protein isoform as well as its intracellular localization, which both warrants further investigations [103].

### 3.2. DDX3

The ATP-dependent DEAD-box RNA helicase DDX3 is involved in multiple steps of RNA metabolism from transcription to translation control, and DDX3 participates in several signaling processes, e.g., innate immune response and Wnt signaling [104,105]. Mechanistically, the helicase activity of DDX3 as well as its interaction with factors of the translation initiation complex may facilitate translation, e.g., by resolving complex structures present in the 5′UTR or by remodeling ribonucleoprotein complexes [106,107,108]. However, the impact of DDX3 on global translation remains controversial [109,110]. Similarly, the expression and function of DDX3 in human cancers is diversified, although DDX3 has been shown to modulate cell adhesion, motility and cancer cell metastasis via the Rac1 pathway [111,112]. Importantly, high expression levels of DDX3 in HNSCC correlated with lymph node metastasis and poor prognosis, and depletion of DDX3 in OSCC cells reduced their proliferation, invasion, and metastatic dissemination in vivo [107]. On the molecular level, DDX3 was shown to act coordinately with the cap-binding complex (CBC) and eukaryotic initiation factor 3 (eIF3) to enhance the translation of Activating transcription factor 4 (ATF4) and other upstream open reading frame (ORF)-containing mRNAs that together modulate EMT programs and promote metastasis [107]. Thus, targeting the novel DDX3-CBC-eIF3 translational complex might be a promising treatment strategy in OSCC.

### 3.3. ELAVL1/HuR

One of the best characterized RBPs in human cancer cells is HuR, the protein product of the embryonic lethal and abnormal vision gene *ELAVL1*. HuR, in contrast to its family members HuB (ELAVL2), HuC (ELAVL3) and HuD (ELAVL4), is ubiquitously expressed in human tissues and is a well-established regulator of post-transcriptional gene expression whose activity is not only controlled by abundance, but also by its subcellular localization [113,114,115]. In detail, under normal cellular and physiological conditions, HuR is primarily located in the nucleus where it can control splicing and alternative polyadenylation [116,117,118]. However, upon exposure to intrinsic or extrinsic stress, it can accumulate in the cytoplasm where it stabilizes and increases the translation of target mRNAs that contain adenine and uridine (AU)-rich elements (AREs) embedded in their 3′UTR [119]. RNA binding is achieved via three RNA recognition motifs (RRMs), whereas translocation is governed by the HuR nucleocytoplasmic shuttling sequence (HNS) located between the second and third RRM [115,120]. Importantly, chronic activation and cytoplasmic localization of HuR can support an inflammatory phenotype, partially due to the stabilization of mRNAs encoding proinflammatory cytokines and enzymes such as transforming growth factor (TGF)-β, tumor necrosis factor (TNF)-α, interferone (IFN)-γ, cyclooxygenase (COX)-2 and others [121,122,123]. Consequently, HuR-dependent inflammation has also been linked to chronic diseases, e.g., pancreatitis, and is thought to underlie HuR’s ability to contribute to many human malignancies including cancer [115,123,124]. In oral cancer it was shown that HuR is localized in the nucleus and cytoplasm of oral cancer cells and tissues, whereas normal oral tissues and cell lines contained HuR almost exclusively in the nucleus [125]. Moreover, loss-of-function experiments revealed an important role for HuR in the transport and stabilization of ARE-containing transcripts, such as cellular oncogene fos (c-FOS), avian myelocytomatosis virus oncogene cellular homolog (c-MYC), and COX-2 mRNAs. Furthermore, HuR was shown to regulate the expression of cell cycle-related proteins (cyclin A, cyclin B1, cyclin D, and cyclin-dependent kinase (CDK) 1) and in the case of CDK1 HuR was able to interact with its mRNA to stabilize it. Not surprisingly, the ability of oral cancer cells to grow in an anchorage-independent manner as well as their motile and invasive capabilities were reduced upon HuR depletion [126]. Analysis of HuR (and COX-2) in OSCC tumor tissues using immunohistochemistry demonstrated that cytoplasmic, but not nuclear HuR immunoreactivity was correlated with COX-2 expression (*p* < 0.025), grade (*p* < 0.014), lymph node (*p* < 0.050), and distant metastases (*p* < 0.025) as well as reduced survival (*p* < 0.023) [127]. Thus, cytoplasmic HuR expression can be considered an independent prognostic marker for poor outcomes in oral cancer, which might, at least partially, be due to its positive effect on COX-2, a pleiotropic cancer gene [128].

### 3.4. ESRP1 and ESRP2

Over the past decade RNA-sequencing technologies and sophisticated analysis pipelines have led to a wave of discoveries regarding the causes and functional relevance of alternative splicing, which has increased the awareness of its potential role in the development and progression of cancer and other diseases [129]. These analyses revealed that more than 95% of protein-coding genes in humans undergo some form of alternative splicing (exon skipping or inclusion of a cassette exon, alternative splice site choice, mutually exclusive exons, intron retention) in a cell type- or condition-specific manner, with at least 80% of these changes altering the protein-coding potential of the transcript or the presence of regulatory sequences in UTRs [130,131]. Importantly, the expansion of the proteome through alternative splicing is an essential driver of cell differentiation and fate decisions and contributes to the ability of cells to respond appropriately to signaling events, e.g., TGF-β-induced EMT [42,132,133]. Two well-known regulators of alternative splicing are the epithelial splicing regulatory proteins 1 and 2 (ESRP1 and ESRP2). Both proteins regulate alternative splicing events associated with epithelial phenotypes of cells, and the expression of both is reduced during EMT [134,135,136]. Mechanistically, ESRP1/2 regulate epithelium-specific splicing by binding of UGG- or GGU-rich sequences that are known as ESRP binding splicing enhancer (EBSE) and ESRP binding splicing inhibitor (EBSI) [42,134]. The location of the EBSE and EBSI elements relative to the alternatively spliced exons determines the outcome, i.e., exon inclusion or skipping. Exon skipping is favoured when ESRP binding sites are located at the 5′ end of, and/or within, the regulated exon, whereas ESRP binding at the 3′ end of regulated exons enhance exon inclusion [42]. Importantly, down-regulation of ESRP1/2 during TGF-β-induced EMT was shown to induce an isoform switch of fibroblast growth factor (FGF) receptors. This sensitizes cells to FGF-2 and, through cooperation with TGF-β, enhances EMT leading to more aggressive phenotypes [137]. On the other hand, enforced overexpression of ESRP1 could suppress malignant phenotypes of colon and breast cancer cells [138,139]. However, whether ESRPs are generally tumor-promoting or inhibiting remains controversial [140]. Intriguingly, a recent study in OSCC revealed a high plasticity of ESRP expression during cancer cell invasion and metastasis [141]. In detail, 49 samples of human HNSCC tissues were examined to detect ESRP1/2 in different stages (normal tissue, dysplasia, carcinoma in situ, invasive carcinoma) and a higher expression level of both proteins was detected in dysplasia and carcinoma *in situ* compared to normal epithelium. Moreover, higher expression of ESRP1/2 was also detected in advanced OSCC and cancer nests in metastatic lymph nodes. In contrast, careful analysis revealed a loss of ESRP1 and ESRP2 expression in cancer cells that penetrated through the basement membrane into the stroma as well as in those cells that invaded from cancer nests into stromal tissues. Hence, down-regulation of ESRP1 and ESRP2 in OSCC might be restricted to cells that acquire a motile phenotype during cancer invasion [141]. These findings highlight the need for single-cell resolved data analyses to obtain a deeper understanding of the molecular changes contributing to cancer metastasis. Moreover, the authors identified distinct mechanisms of action of ESRP1 and ESRP2 in oral cancer cells. First, depletion of ESRP1 principally inhibited cell motility via regulation of actin dynamics. This was partially achieved by inclusion of exon 3b in alternative splicing of Rac1 mRNA, thereby increasing expression of the Rac1b isoform upon ESRP1 depletion. Rac1b modulated actin dynamics to induce formation of long filopodia and augment cell motility. In contrast, depletion of ESRP2 strongly repressed E-cadherin mRNA and protein expression. Of note, levels of ZEB1 and ZEB2, two EMT-associated transcription factors that repress E-cadherin expression, were elevated upon ESRP2 silencing. However, it remains to be elucidated how ESRP2 is able to represses ZEB1/2 expression and whether splicing-independent mechanisms, e.g., regulation of miRNA biogenesis might underlie ESRP2 function in oral cancer cells [141]. Another interesting function of ESRP1/2 in OSCC was recently described in a study focusing on the oncogenic roles and biogenesis of a circular RNA (circRNA) called circUHRF1. The authors could show that this circRNA is able to promote proliferation, migration, invasion, and EMT in vitro as well as oral cancer cell growth in vivo by sponging miR-526b-5p and lifting c-MYC expression [142]. Of note, the circularisation and biogenesis of circUHRF1 was accelerated by ESRP1 binding to the flanking introns thereby enforcing a circUHRF1/miR-526b-5p/c-MYC/TGF-β1/ESRP1 feedback loop. Given the controversial roles of ESRPs and the spatially fine-tuned expression of both proteins in OSCC tissues as mentioned before, it would be interesting to investigate, if this axis contributes to non-cell autonomous mechanisms of EMT and metastasis through the secretion of TGF-β into the microenvironment.

### 3.5. IGF2BP3

The mammalian Insulin-like growth factor 2 mRNA-binding protein (IGF2BP) family comprises of three RBPs. Two members of the family, IGF2BP1 and IGF2BP3, are de novo synthesized in various human cancers and function as bona fide oncofetal proteins [143]. All family members share a conserved domain structure including two RRMs at the N-terminus and four C-terminal heterogeneous nuclear ribonucleoprotein (hnRNP) K homology (KH) domains, the latter being essential for RNA-binding and thereby determine the mainly cytoplasmic, granular distribution pattern of all three proteins [144]. Despite their high degree of structural similarity, the IGF2BP family members exhibit quite different expression patterns and exhibit distinct RNA-binding properties and presumably associate with variable target transcripts that might contain the putative consensus binding motif CAUH (H = A, U, or C) [145]. Importantly, all paralogues were shown to control the turnover, translation and/or transport of their target transcripts and there is the assumption that all IGF2BPs direct mRNA fate via cytoplasmic RNPs in which IGF2BPs might associate with other RBPs, mainly or exclusively in an RNA-dependent manner [144]. Functional studies over the last decades revealed that IGF2BPs modulate the expression of genes implicated in the control of cell proliferation, survival, chemo-resistance, and metastasis. Consistently, the expression of IGF2BP family members was reported to correlate with an overall poor prognosis and metastasis in various human cancers [143,146]. In OSCC, several studies specifically identified IGF2BP3 expression to be upregulated in oral squamous cell carcinoma [147,148,149,150,151,152,153]. However, all studies relied on a non-paralogue-specific antibody and some results have to be considered with great caution [146]. Nevertheless, several studies established IGF2BP3 as a predictor of lymph node status and metastasis and its expression was shown to correlate with an overall poor prognosis in OSCC [147,148,149,150,151,152,153]. On the cellular level, it has been reported that IGF2BP3 is specifically overexpressed at the invasive front of invasive OSCC cells and its depletion reduced the invasive capacity of oral cancer cells and impaired tumor growth in a mouse xenograft model [148,154]. A key target of IGF2BP3 in OSCC seems to be podoplanin (PDPN). PDPN is also specifically expressed at the invasive front of tumors and IGF2BP3 regulates the PDPN expression by binding to the 3′UTR of the PDPN mRNA, thereby stabilizing the transcript [148]. Notably, both IGF2BP3 and PDPN expression was shown to correlate with lymph node metastasis in OSCC patients and IGF2BP3 downregulation inhibited invadopodia formation, extracellular matrix degradation, and tumor growth and invasiveness [148,154]. In addition, the expression of both IGF2BP3 and PDPN together was associated with bone invasion and the number of osteoclasts in patients with OSCC and IGF2BP3 or PDPN depletion inhibited the invasive capacity of OSCC cells in a three-dimensional culture system, tumorigenesis, and regional bone destruction in a xenograft mouse model [155]. Furthermore, IGF2BP3 was investigated in preoperative biopsy material and appeared to be predictive of perineural invasion in patients with OSCC and IGF2BP3 status was an independent predictor of death of patients with OSCC [156]. Although the detailed mechanisms that drive IGF2BP3 expression in oral cancer remain to be determined, a recent study identified epidermal growth factor (EGF) as an inducer of IGF2BP3 and PDPN expression [157]. However, additional studies are required to comprehensively map the RNA and protein interactome of IGF2BP3, and to characterize the function of the other IGF2BP family members in oral cancer.

### 3.6. LIN28B

The *LIN28B* gene belongs to the lin-28 family that was initially discovered in *Caenorhabditis elegans* where it controls developmental timing [158]. In humans, the family is comprised of two members, namely LIN28A and LIN28B that are both able to bind RNA via their cold-shock domain and the two Cys-Cys-His-Cys (CCHC)-type zinc finger domain. In addition, LIN28B contains a nuclear and nucleolar localization signal responsible for its nuclear accumulation, whereas LIN28A is primarily located in the cytoplasm [159]. The difference in their intracellular localization underlies their partially overlapping, yet largely different cellular functions and mechanisms. For example, both paralogues were shown to inhibit let-7 miRNA biogenesis, although by distinct molecular mechanisms [160]. A huge body of evidence suggests that Lin28 proteins significantly contribute to pluripotency, reprogramming and tumorigenesis. They regulate cancer cell proliferation, metabolism, resistance to radiotherapy as well as chemotherapy and induce cancer stem cell (CSC) formation. Importantly, CSCs are associated with cancer development, progression, metastasis, recurrence and therapy resistance in HNSCC [159]. Intriguingly, Lin28A and Lin28B were found to be increased in OSCC and Kaplan-Meier analysis showed that patients with high Lin28B but not Lin28A expression had lower overall survival rates than those with low Lin28B expression [161]. In the line with this, stable overexpression of Lin28B in oral cancer cells promoted cell migration, invasion, colony formation, and tumor growth in vivo. While the precise molecular mechanism of Lin28B was not thoroughly investigated, the authors observed an increase of diverse and well-known regulators of cancer phenotypes, e.g., interleukin-6 (IL-6), high-mobility group AT-hook 2 (HMGA2), snail family transcriptional repressor 1 (SNAI1), TWIST, vascular endothelial growth factor (VEGF), and baculoviral IAP repeat containing 5 (BIRC5) [161]. Nevertheless, the clinical relevance of Lin28B could be confirmed by other studies. For example, Wang and colleagues also found that high Lin28B expression in OSCC was significantly associated with short overall survival, and a multivariate survival analysis revealed that Lin28B expression status was a critical independent prognostic marker for overall survival of OSCC patients [162]. Notably, the authors could show that Lin28B abundance was associated with tumor size as well as cervical lymph nodes metastasis [162]. Another recent study used tumor tissue samples and matched adjacent non-cancerous tissues as well as lymph node metastatic lesions from OSCC patients and measured Lin28B transcript levels via quantitative real-time polymerase chain reaction (qRT-PCR) [163]. Again, higher Lin28B levels were present in tumors compared to normal tissues with an additional increase seen in metastatic samples. Depletion of Lin28B attenuated carcinogen-induced proliferation, migration, and invasiveness of oral cancer cell lines in vitro, yet the downstream targets of Lin28B remain elusive [163]. In order to investigate Lin28B and its role in OSCC cancer stem cells, Chien et al. analyzed and compared the expression levels of Lin28B in nine pairs of tumorous and non-tumorous tissues of oral cancer patients using immunohistochemistry as well as qRT-PCR, confirming the upregulation in transformed tissues [164]. Moreover, the authors found that cells expressing CSC markers, i.e., cluster of differentiation (CD) 44 and aldehyde dehydrogenase 1 (ALDH1), had high mRNA expression levels of Lin28B. Subsequent functional analyses revealed that the Lin28B/Let-7 pathway positively regulates POU class 5 homeobox 1 (POU5F1) and SRY-box transcription factor 2 (SOX2) expression in OSCC, thereby inducing a reprogramming-like phenomenon, switching non-CSCs to CSCs with tumor-initiating and self-renewal properties. By suppressing let-7, Lin28B enhances the expression of AT-rich interaction domain molecule 3B (ARID3B) and HMGA2 proteins, which directly regulate POU5F1 and SOX2 promoter activity, respectively [164]. Taken together, these studies highlight the importance of Lin28B in OSCC metastases and establish this RBP as an important prognostic marker as well as therapeutic target in oral cancer.

### 3.7. METTL3-METTL14 Complex

In the last couple of years, chemical modifications in RNA—also referred to as ‘RNA epigenetics’ or the ‘epitranscriptome’—have been extensively studied and their dynamic distribution as well as the impact of modified bases on the metabolism and fate of both coding and non-coding transcripts has been revealed [165,166,167]. N6-Methyladenosine (m6A) accounts for the most abundant internal modification in eukaryotic mRNA where it is specifically found in the consensus DRACH motif (where D = A/G/U, R = A/G, and H = A/C/U). It was shown to control gene expression in diverse physiological and pathophysiological processes by tuning RNA stability, splicing, and translation [168]. The vital roles of m6A in diverse biological processes are dependent on many RBPs with catalytic and non-catalytic roles as readers, writers and erasers of m6A [169,170]. The methyltransferase-like 3 and 14 (METTL3 and METTL14) proteins are part of the main RNA methyltransferase complex, which is comprised of METTL3, METTL14, and Wilms’ tumor 1-associated protein (WTAP). Importantly, METTL3 is the enzymatic component of the complex whereas METTL14 acts as an allosteric activator and plays a structural role critical for substrate recognition and their genetic ablation in mouse embryonic stem cells led to a loss of 99% of all m6A in poly(A) RNA [171,172,173,174]. Of note, oncogenic roles for METTL3 and METTL14 have been described in several human cancers including acute myeloid leukemia (AML), lung and liver cancer in which they increase the expression of oncogenes, e.g., MYC, SNAI1, and EGFR, or enhance the degradation of tumor suppressors, e.g., suppressor of cytokine signaling 2 (SOCS2) to drive tumor growth signaling. However, the role of the METTL3-METTL14 complex might be dependent on the cancer type, and also tumor suppressive functions have been described [165,168]. However, the contributions of m6A as well as the respective reader, writer and eraser proteins for the development and progression of OSCC are not well understood yet. Only recently the expression profiles of 13 m6A-related genes in 317 OSCC and 32 normal samples from The Cancer Genome Atlas (TCGA) database had been analyzed and found a significantly higher expression of eight genes, including METTL3 and METTL14, in tumor tissues. Furthermore, an increase of m6A levels in total tumor RNA could be detected using an antibody-based quantification kit [175]. The upregulation of METTL3 in oral cancer was also seen in two additional studies that also report a correlation of METTL3 expression with poor prognosis of OSCC patients [176,177]. Both studies detected similar cellular phenotypes, i.e., METTL3 promoted the proliferation, self-renewal, invasion, and migration of OSCC cells in vitro, as well as tumor growth and metastasis in vivo [176,177]. In addition, a genetically modified mouse model revealed an essential role of Mettl3 in chemical-induced oral carcinogenesis [176]. However, both studies discovered varying mechanisms, which are not mutually exclusive and might simply reflect the overall diverse molecular targets and modes of action of METTL3 and/or m6A. In detail, Zhao and colleagues performed methylated RNA immunoprecipitation sequencing (MeRIP-seq) and identified the c-MYC mRNA as a m6A-modified transcript whose stability is positively regulated by the m6A writer METTL3 and the YTH domain family member 1 (YTHDF1) protein acting as m6A reader [177]. Hence, the METTL3/m6A/YTHDF1/c-Myc axis might be a novel and promising target for OSCC therapy. Alternatively, METTL3-catalysed methylation of epigenetic regulators might also represent a novel targeting option. Specifically, Liu and colleagues could show that METTL3 is responsible for the deposition of m6A marks in the 3′UTR of the B lymphoma Mo-MLV insertion region 1 homolog (BMI1) mRNA [176]. BMI1, a CSC marker and component of the Polycomb Repressive Complex 1 (PRC1) that is responsible for chromatin remodeling and epigenetic gene silencing, has been previously shown to promote chemoresistance and metastasis in HNSCC and plays an important role in the progression and prognosis of OSCC [178]. Here, METTL3, in cooperation with the m6A reader IGF2BP1, enhanced the translation of BMI1 in oral cancer cells, which is thought to underlie the observed cellular phenotypes upon METTL3 depletion or overexpression, although this remains to be formally proven [176,179]. Furthermore, the crosstalk between METTL3-dependent changes in epitranscriptome and their impact on the epigenetic modifications found in the oral cancer genome, including a potentially altered chromatin organization, need to be analyzed in more detail in the future. Nevertheless, the m6A pathway and its catalytic components, e.g., METTL3, represent interesting and druggable targets to altered RNA metabolism in oral cancer cells.

In summary, several individual RBPs have been identified as important regulators of oral cancer cell phenotypes as well as prognostic markers in OSCC. The herein presented examples highlight the broad variety of RBP functions and mechanisms of activation and establish these RBPs as clinically relevant targets in OSCC. Additional RBPs with a role in oral cancer cell migration, invasion, and metastasis are summarized in Table 1. In the following paragraph we aim to provide a brief overview about the recently developed targeting strategies.

## 4. Therapeutic Targeting Options for RBPs Involved in Oral Cancer Progression

In this final chapter we will focus on small molecules and chemistry-based strategies to target OSCC-associated RBPs. We will not discuss other genetic or non-genetic option, e.g., gene therapy, small interfering RNA (siRNA), circular RNAs, aptamers, or RNA-targeted therapies, which were recently discussed elsewhere [188,189].

Small molecules have been developed to target disease-related proteins like ion channels, G-protein-coupled receptors, nuclear receptors, and kinases and these molecules are commonly and successfully used as therapeutics in clinical applications [190]. However, the target space (~700 proteins) of small molecules with regulatory approval is rather limited and currently accounts for less than 0.5% of the human proteome. Similar to most transcription factors, RBPs are generally considered to be difficult to target and only a limited number of small molecules have been discovered that inhibit the function of RBPs involved in oral cancer progression (Table 2).

A widely applied strategy to interfere with the function of an RBP is to modulate the interaction with its key coding or non-coding target RNA that is thought to be essential for the disease-related function of the RBP. This can be achieved either via targeting the RNA or the RBP directly. RNA-targeting strategies, which include nucleotide-based agents that target unstructured regions of RNA as well as small molecules that bind directly to structured RNAs are showing promising clinical success and have been discussed elsewhere [188,224,225]. In contrast, RBP-targeting approaches remain largely underdeveloped. Major hurdles that make it challenging to obtain small molecules that specifically and effectively inhibit RBPs through a competitive binding mechanism are: (i) the strong electrostatic attraction between the negatively charged RNA and the positively charged binding domains of RBPs, (ii) the large interacting surface involved in many protein–RNA interactions, and (iii) the commonly used and conserved structures of the RBDs that are present in many RBPs and could cause off-target binding of the small molecules. Hence, most small molecule inhibitors of RBPs have weak, micromolar-range inhibitory potency, and their proteome-wide selectivity is often unknown. Of note, many of the RBP-targeting small molecules contain ring systems and off-target binding to other proteins is very likely [226]. For example, the natural product (-)-gossypol was identified as a high-affinity binder and inhibitor of MSI1 [221]. However, gossypol was also shown to bind to the really interesting new gene (RING) domain of mouse double minute 2 (MDM2) as well as to inhibit the tyrosine kinase activity of wild-type and mutant EGFR [227,228,229]. Moreover, the LIN28 inhibitor compound 1632 was shown to have micromolar binding affinity for the bromodomains of bromodomain-containing protein 4 (BRD4) and CREB-binding protein (CREBBP) [212]. Similar off-target effects might be found for other RBP inhibitors and might be responsible for their biological activities and observed effects on cell growth and other cancer phenotypes. Hence, more rigorous binding and specificity analyses should be implemented to obtain small molecules with high affinity and selectivity for individual RBPs. Furthermore, novel screening approaches and targeting concepts should be developed and tested considering the following points:

First, small molecule inhibitors of RBPs are commonly developed from hits initially identified in high-throughput screen and the chemical space covered in the screening libraries will therefore determine the biological activity and specificity of the hit compound, and ultimately the success of the screen. Thus, more complex and diverse libraries should be used in order to retrieve compounds with new biological activities against RBPs. To this end, libraries should include both protein-targeting and RNA-targeting molecules to ensure that RBP-RNA interactions are comprehensively interrogated. Second, the structures of RBPs, both in the presence and absence of their RNA substrate(s) should be systematically solved and catalogued. This will allow subsequent hit optimization by structure-activity relationship analysis and will ultimately shift the discovery of RBP inhibitors from screening-based approaches towards rational drug design-based approaches.

Second, the screening goals should be re-evaluated. For example, instead of aiming to identify small molecules that compete with RNA for binding to RBPs, it might be possible, e.g., by leveraging structural information, to identify allosteric binding pocket in RBPs that could be targeted by small molecules to block functional relevant conformational changes in RBPs. Additionally, unbiased screening to identify high-affinity binders irrespective of their binding sites on the RBP might enable the development of novel, chimeric molecules, so called proteolysis targeting chimeras (PROTACs) that could be used to induce proteasomal degradation of the target RBP [230].

Another aspect that should be considered in the design of future screening and targeting strategies is the dual functioning of some RBPs. For example, RBPs might not only act by modulating RNA metabolism, but might also bind to DNA and other proteins, or contain (pseudo)kinase and RING domains, which suggest non-canonical, potentially RNA-independent functions and novel options to modulate the activity of RBPs [231,232,233,234]. Furthermore, several RNA-dependent protein complexes that might comprise of direct and indirect RNA binders have been recently identified [235]. Disruption of these complexes by interfering with protein-protein interactions could offer an alternative targeting approach [236].

Last but not least, functional genetic screens could be used to identify synthetic vulnerabilities that could be exploited to target cancer cells that show an altered expression or mutations in RBP genes [237,238,239,240].

## 5. Conclusions

RNA-binding proteins comprise a huge and diverse class of important regulators of cellular plasticity and homeostasis. Their significant contribution to disease phenotypes has been broadly established. However, the therapeutic targeting of RBPs remains challenging. Thus, future research endeavors should try to identify disease-specific RBP-dependencies, explore indirect (synthetic) vulnerabilities, and develop innovative targeting approaches or leverage existing concepts to make this class of proteins accessible for RBP-directed clinical interventions.

## Figures and Tables

**Table 1:** Additional RBPs with a role in oral cancer cell migration, invasion, and metastasis.

RBP	Function & Mechanism	Reference
***HuD***	expression associated with lymph node metastasis and mode of invasion; depletion reduced invasiveness of cells and transcript levels of VEGF-A, VEGF-D, matrix metalloproteinase-2 (MMP-2) and MMP-9	[180]
***HNRNPL***	overexpressed in OSCC compared to normal oral mucosa tissue; depletion reduced proliferation, viability, migration and in vivo growth; regulates SRSF3 RNA expression & alternative splicing of exon 4	[181]
***Musashi 1*** ***(MSI1)***	higher expression in higher stages and poorly differentiated tumors; independent prognostic marker of overall and disease-free survival	[182]
	higher MSI1 mRNA level in OSCC compared to matched healthy tissue; depletion reduced proliferation, invasion, migration, and in vivo growth; might regulate c-MYC expression and STAT3 activation	[183]
***Quaking*** ***(QKI)***	lower expression in OSCC compared to normal mucosal samples; overexpression reduced stemness, tumor growth and metastasis; depletion enhanced Sox2 mRNA stability and expression	[184]
	low expression in HNSCC resulted in shorter overall survival; targeted by miR-200 family; knockdown induced EMT and enhanced migration and invasion in vitro and tumor growth and metastasis *in vivo*;	[185]
***SRSF3***	up-regulated in moderate or severe dysplasia tissues compared with normal oral mucosal tissues, and higher grade cancers express more SRSF3; expression correlated with lymph node metastasis and its depletion reduced EMT-related genes SNAI2 and N-cadherin	[186]
***La***	overexpressed in OSCC tissue compared to normal epithelial tissue; depletion inhibited proliferation, migration and invasion; knockdown reduced MMP-2 and β-catenin protein expression	[187]

**Table 2:** Inhibitors of RBPs involved in oral cancer progression.

Target RBP	Compound	Mode of Action	Reference
***ADAR1***	8-Azaadenosine	Inhibits RNA editing activity	[191]
***DDX3***	RK-33	Inhibits helicase or ATPase activity	[192,193,194]
	NZ51	Inhibits helicase activity	[195,196]
	FE15/FE87/FE98/FE109	Inhibit the ATPase activity	[197,198]
	Compounds 1 & 3	Target the RNA binding site	[199]
	Compounds 6 & 8	Inhibit helicase and ATPase activity	[199]
	Ketorolac salt	Reduces DDX3 expression and inhibits ATPase activity	[200]
***HuR***	MS-444, Okicenone, Dehydromutactin	Interfere with formation of HuR dimers and thereby RNA binding	[201]
	Quercetin, b-40	Inhibit HuR:ARE (TNF-α) complex formation	[202]
	Mitoxantrone	Inhibit HuR:ARE (TNF-α) complex formation	[203]
	dihydrotanshinone-I	Inhibits binding of HuR to several RNAs	[204]
	Compound 10	Disrupts HuR oligomerization	[205]
	CMLD-2	Inhibits binding of HuR to ARE-containing target RNAs (Bcl-2, MSI1 and XIAP)	[206]
	Azaphilone-9	Inhibits HuR:ARE interaction by competitive binding in the RNA-binding cleft	[207]
***IGF2BP3***	d-ICD	Inhibits IGF2BP3 expression	[208]
	JQ1, iBET	Inhibit IGF2BP3 expression	[209,210]
***LIN28***	Compound 1	Inhibits LIN28–pre-let-7 interaction	[211]
	Compound 1632	Inhibits LIN28–pre-let-7 interaction	[212]
	6-hydroxy-DL-DOPA, SB/ZW/0065	Inhibit LIN28–pre-let-7 interaction	[213]
	LI38 (TPEN), LI71	Inhibit LIN28-mediated oligouridylation of let-7	[214]
	KCB170522, Luteolin	Inhibit LIN28–pre-let-7 interaction	[215]
	CCG-233094, CCG-234459	Inhibit LIN28–pre-let-7 interaction	[216]
***METTL3***	ribofuranuronic acid analogues of adenosine, adenosine analogue with a tetrahydropyran ring	Competitors of S-adenosyl-L-methionine (SAM) for METTL3 binding	[217]
***MSI1***	Inhibitor #1-3	Inhibit RNA binding activity of MSI1/2	[218]
	Ro 08-2750	Inhibits RNA binding activity of MSI1/2	[219]
	Oleic acid	Induces a conformational change that prevents RNA association	[220]
	(-)-gossypol	Interacts with RNA binding pocket and blocks MSI1-RNA interaction	[221]
***La***	HBSC-11	Reduced La mRNA and protein levels	[222]
***SRSF3***	Palmitic Acid	Increases neddylation and degradation of SRSF3 protein	[223]

## Abbreviations

A	Adenosine
ADAR1	Adenosine deaminase acting on RNA 1
ALDH1	Aldehyde dehydrogenase 1
AML	Acute myeloid leukemia
ARE	(AU)-rich element
ARID3B	AT-rich interaction domain molecule 3B
ATF4	Activating transcription factor 4
BIRC5	Baculoviral IAP Repeat Containing 5
BMI1	B lymphoma Mo-MLV insertion region 1 homolog
BRD4	Bromodomain-containing protein 4
CBC	Cap-binding complex
CD	Cluster of differentiation
CDK	Cyclin-dependent kinase
CDKN2A	Cyclin dependent kinase inhibitor 2A
c-FOS	cellular oncogene fos
circRNA	circular RNA
c-MYC	Avian myelocytomatosis virus oncogene cellular homolog
COX	Cyclooxygenase
CREBBP	CREB-binding protein
CSC	Cancer stem cell
DDX3	DEAD Box Protein 3
EBSE	ESRP binding splicing enhancer
EBSI	ESRP binding splicing inhibitor
EGF	Epidermal growth factor
EGFR	Epithelial growth factor receptor
eIF3	eukaryotic initiation factor 3
ELAVL	Embryonic lethal and abnormal vision gene
EMT	Epithelial-to-mesenchymal transition
ESRP	Epithelial splicing regulatory protein
FGF	Fibroblast growth factor
HMGA2	High-mobility group AT-hook 2
hnRNP	heterogeneous nuclear ribonucleoprotein
HNRNPL	Heterogeneous nuclear ribonucleoprotein L
HNS	HuR nucleocytoplasmic shuttling sequence
HNSCC	Head and neck squamous cell carcinoma
HPV	Human papilloma virus
HuB	Hu antigen B
HuC	Hu antigen C
HuD	Hu antigen D
HuR	Human antigen R
I	Inosine
IFN	Interferone
IGF2BP	Insulin-like growth factor 2 mRNA binding protein
IL-6	Interleukin 6
JAK	Janus kinase
KH	K homology
La	Small RNA binding exonuclease protection factor La
lncRNA	long non-coding RNA
M6A	N6-Methyladenosine
MDM2	Mouse double minutes 2
MeRIP-seq	Methylated RNA immunoprecipitation sequencing
METTL	Methyltransferase-like
miRNA	microRNA
mRNA	messenger RNA
MSI1	Musashi1
mTOR	mammalian target of rapamycin
ncRNA	non-coding RNA
ORF	Open reading frame
OSCC	Oral squamous cell carcinoma
PDPN	Podoplanin
PI3K	Phosphatidylinositol-3-kinase
POU5F1	POU class 5 homeobox 1
PRC1	Polycomb repressive complex 1
PROTAC	Proteolysis Targeting Chimeras
QKI	Quaking
RB1	Retinoblastoma-associated protein 1
RBD	RNA-binding domain
RBL1/2	Retinoblastoma-like protein 1
RBP	RNA-binding protein
RING	Really Interesting New Gene
RISC	RNA-induced silencing complex
RRM	RNA recognition motifs
SNAI1	Snail family transcriptional repressor 1
SOCS	Suppressor of cytokine signaling
SOX2	SRY-Box transcription factor 2
SRSF3	Serine and arginine rich splicing factor 3
STAT	Signal transducer and activator of transcription
TCGA	The Cancer Genome Atlas
TGF	Transforming growth factor
TNF	Tumor necrosis factor
TP53	Tumor protein 53
TTP	Tristetraprolin
UTR	Untranslated region
VEGF	Vascular endothelial growth factor
WTAP	Wilms’ tumor 1-associated protein
YTHDF1	YTH domain family member
ZEB	Zinc finger E-box binding homeobox
